# *Pseudomonas sivasensis* 2RO45 inoculation alters the taxonomic structure and functioning of the canola rhizosphere microbial community

**DOI:** 10.3389/fmicb.2023.1168907

**Published:** 2023-05-05

**Authors:** Joanna Świątczak, Agnieszka Kalwasińska, Attila Szabó, Maria Swiontek Brzezinska

**Affiliations:** ^1^Department of Environmental Microbiology and Biotechnology, Nicolaus Copernicus University in Toruń, Toruń, Poland; ^2^Department of Aquatic Sciences and Assessment, Swedish University of Agricultural Sciences, Uppsala, Sweden

**Keywords:** PGPR, *Pseudomonas*, rhizosphere diversity, metabolic functions, canola microbiome

## Abstract

Inoculation with plant growth-promoting rhizobacteria (PGPR) is an eco-friendly sustainable strategy for improving crop productivity in diverse environments under different conditions. Our earlier study demonstrated that *Pseudomonas sivasensis* 2RO45 significantly stimulated canola (*Brassica napus* L. var. *napus*) growth. The aim of the present study was to investigate the structural and functional dynamics in the canola rhizosphere microbiome after inoculation with PGPR *P. sivasensis* 2RO45. The results based on alpha diversity metrics showed that *P. sivasensis* 2RO45 did not significantly alter the diversity of the native soil microbiota. However, the introduced strain modified the taxonomic structure of microbial communities, increasing the abundance of plant beneficial microorganisms, e.g., bacteria affiliated with families *Comamonadaceae*, *Vicinamibacteraceae*, genus *Streptomyces*, and fungi assigned to *Nectriaceae, Didymellaceae, Exophiala, Cyphellophora vermispora*, and *Mortierella minutissima*. The analysis of community level physiological profiling (CLPP) revealed that microbial communities in the *P. sivasensis* 2RO45 treated canola rhizospheres were more metabolically active than those in the non-treated canola rhizosphere. Four carbon sources (phenols, polymers, carboxylic acids, and amino acids) were better metabolized by the microbial communities from the rhizosphere of plants inoculated with the *P. sivasensis* 2RO45 than non-inoculated canola rhizospheres. Based on the community-level physiological profiles, the functional diversity of the rhizosphere microbiome was altered by the *P. sivasensis* 2RO45 inoculation. Substrate utilization Shannon diversity (H) index and evenness (E) index were significantly increased in the treated canola plants. The study provides new insight into PGPR-canola interactions for sustainable agriculture development.

## 1. Introduction

The rhizosphere is a hotspot around the roots where numerous important processes related to the nutrition, growth, and fitness of plants occur (Sugiyama et al., 2014; Zuluaga et al., 2021). Rhizosphere microorganisms, such as PGPR, are regarded as prominent components of sustainable agriculture due to their positive influence on plant growth through alleviation of biotic and abiotic stresses, providing nutrients and secretion of phytohormones (Zuluaga et al., 2021).

The native rhizosphere microbial communities are altered by numerous interactions, including interfering in root exudation patterns, exchange of genetic material, and transformation of nutrients. Rhizosphere microbiome may be also modified by PGPR inoculation (Bhattacharyya and Lee, 2016). Therefore, before the use of PGPR as bioinoculants in the field, it is necessary to determine the changes occurring in the resident soil microorganisms structure after PGPR inoculation. It was reported that PGPR inoculation can lead to transient or even permanent alterations in the abundance of microbial communities and their function, which can finally contribute to promoting plant growth and its fitness (Bhattacharyya and Lee, 2016; Kong and Liu, 2022).

Because rhizosphere microbial communities are important for plant growth promotion; an increasing number of studies pay more attention to the next generation sequencing (NGS) methods to better understand how PGPR inoculation affects the structure of rhizosphere microbiomes (Sugiyama et al., 2014). Moreover, it was reported that the effect of PGPR inocula on the functional diversity of the native rhizosphere microbial community should receive more attention (Di Salvo et al., 2018a). Some researchers analyzed the changes in microbial communities’ metabolic activity and function after PGPR inoculation using the community-level physiological profiling (CLPP) method. However, the analyses on different crops, such as rice, tomato, maize and wheat have been performed (Naiman et al., 2009; de Salamone et al., 2010; Di Salvo et al., 2018a; Zuluaga et al., 2021).

Our earlier results showed that the bacterization of canola (*Brassica napus* L. var. *napus*) seeds with *Pseudomonas sivasensis* 2RO45 significantly promoted growth of plant (Świątczak et al., unpublished). This study aimed to determine whether the bacterization of canola seeds with *P. sivasensis* 2RO45 alters the structural and functional diversity of microbial communities in the rhizosphere. The taxonomic structure changes in bacterial and fungal communities were evaluated using NGS, while the function of the microbial community was investigated by CLPP method using Biolog EcoPlates. The analyzes of interactions between canola plants and their associated microbiota after PGPR inoculation can provide new approaches for canola growth management.

## 2. Materials and methods

### 2.1. Rhizobacterium *Pseudomonas sivasensis* 2RO45

The *Pseudomonas sivasensis* 2RO45 was originally isolated from the canola (*Brassica napus* L. var. *napus*) rhizosphere taken from the field in Ostroda, Poland (53°41′38″N 19°57′58″E). The 2RO45 was selected based on its PGP traits, and its ability to promote canola growth under sterile and non-sterile conditions. The 2RO45 was able to produce indole-3-acetic acid (IAA), sequester siderophores, solubilize phosphates, and to produce 1-aminocyclopropane-1-carboxylic acid (ACC) deaminase.

### 2.2. Experimental design and rhizospheric soil sampling

*Pseudomonas sivasensis* 2RO45 cells were harvested in LB broth (10 ml) with 0.05 g carboxymethyl cellulose (CMC) to yield 10^8^ colony forming units (CFU)/ml. Seeds of canola were sterilized by soaking for 30 min in 1% sodium hypochlorite, followed by three rinses with sterile distilled water and incubation with 2RO45 inoculum for 30 min in a shaker incubator. Control seeds were incubated in LB broth (10 ml) with 0.05 g CMC under the same conditions (Rudolph et al., 2015). Four seeds were sowed in pots containing soil taken from the field in Ostroda, Poland (53°41′38″N 19°57′58″E). The bacterized and non-bacterized rhizospheres samples (total of 24 samples) were collected from the canola roots by scraping the adhering soil after 0 (T0), 22 (T22), and 44 days (T44). Four replicate pots were maintained for each time-point (Supplementary Figure 1). The “time” was considered as a main factor for establishing the groups of samples, in which the changes in microbiota structure and function were evaluated.

### 2.3. Colonization efficiency of *Pseudomonas sivasensis* 2RO45

To test if P. *sivasensis* 2RO45 can colonize the canola rhizosphere, sterilized canola seeds were inoculated with 2RO45 (10^8^ CFU/ml) under sterile soil conditions. Canola plants were maintained in a growth chamber in a day-night cycle of 16 h light (100 μmol/m2/s) and a temperature of 22°C. Root samples were collected after 0 (T0), 22 (T22), and 44 days (T44). The plant roots were vortexed for 30 min in PBS solution (Ke et al., 2019) and the number of P. *sivasensis* 2RO45 were counted on PCA medium after incubating at 28°C for 48 h.

### 2.4. DNA extraction and sequencing

Rhizosphere samples collected from the replicate pots of each treatment were pooled separately (Supplementary Figure 1; Bhattacharyya et al., 2018) and total genomic DNA was extracted from 0.25 g of fresh rhizosphere samples using a DNeasy Power Soil Kit (Qiagen, Hilden, Germany), following the manufacturer’s protocol. DNA concentration was determined using Qubit dsDNA HS Assay Kit (Thermo Fisher Scientific, Waltham, MA, USA). Then, each sample was sent for (NGS) at the University of Łódź (Biobank), Poland.

Bacterial and fungal communities were determined by the amplification of V3-V4 hyper-variable regions of the 16S rRNA gene and Internal Transcribed Spacer (ITS2), respectively. For bacteria, the following primers were used: 5′ CCTACGGGNGGCWGCAG (forward) and 5′ GACTACHVGGGTATCTAATCC (reverse) (Klindworth et al., 2013). For fungi, a forward primer was used: 5′GCATCGATGAAGAACGCAGC (ITS3, without overhangs) with the reverse primer 5′ TCCTCCGCTTATTGATATGC (ITS4, without overhangs) (White et al., 1990). The methodology for library preparation followed the protocol available from Illumina Support Center (ISC) with a slight modification (2 × Phanta Max Master Mix, Vazyme Biotech, Nanjing City, China was applied instead of Kapa Hifi Hot Start Ready mix). The products were verified after each PCR using the electrophoretic separation. Libraries were normalized based on band luminescence intensity on a 1.5% agarose gel and pooled. Sequencing was performed on a MiSeq (Illumina, San Diego, CA, USA) using MiSeq Reagent Kit v2 (500-cycles) 2 × 250 bps paired-end format.

### 2.5. Amplicon sequence analysis

In the present study, the merging of sequence sets was performed, and the analysis of 16S rRNA amplicons was carried out using mothur v1.44.3 (Schloss et al., 2009). The quality processing, taxonomic assignments, and operational taxonomic unit (OTU) picking were done according to the MiSeq SOP of mothur (Kozich et al., 2013) with a 97% similarity threshold. The ‘make.contigs’ command was executed with a deltaq value of 10, and the analysis excluded ambiguous base calls and reads shorter than 300 nt or longer than 500 nt. The end of the sequences was trimmed to remove the primers using trim.seqs (pdiffs = 2, checkorient = T). The pre.cluster command was utilized for denoising, and chimeric reads were filtered out using VSEARCH in mothur. The sequence set was further processed by discarding the singleton reads as per Kunin et al. (2010). The read alignment and taxonomic assignment were carried out using the ARB-SILVA SSU Ref NR 138 reference database (Quast et al., 2013) with a minimum bootstrap confidence score of 80. Reads assigned to non-primer-specific taxonomic groups (“Chloroplast,” “Mitochondria,” and “unknown”) were excluded from the dataset. Finally, a random subsampling was performed based on the sample with the lowest sequence number.

Fungal ITS analysis was performed through the following steps. The merging and quality filtering of sequences were carried out using mothur, as described for the 16S rRNA gene amplicons. The ITS2 region was extracted from the sequences with the ITSx 1.1-beta software (Bengtsson-Palme et al., 2013) based on the findings of Nilsson et al. (2010). Taxonomic assignment was performed using mothur’s classify.seqs (cutoff = 80) and the UNITE v8.3 database (Abarenkov et al., 2020) as reference. Sequences that were not assigned to any fungal phyla were discarded from the amplicon set with the remove.lineage (taxon = k__Fungi_unclassified) command of mothur. Finally, the clustering of ITS2 reads into OTUs was performed using VSEARCH with a 97% similarity threshold.

The sequences were uploaded to the GenBank-Sequence Read Archive under Bioproject PRJNA876229.

### 2.6. Functional potential of microbial communities

The effect of *Pseudomonas sivasensis* 2RO45 inoculation on the microbial community function and diversity in the canola rhizosphere were analyzed by the CLPP method using Biolog EcoPlates (Biolog Inc., Hayward, CA, USA). Rhizosphere samples (10 g) were collected after 44 days of *P. sivasensis* 2RO45 inoculation. Samples were added to 90 ml of 0.85% sterile saline solution and shaken for 30 min at 200 rpm (Sun et al., 2010). Samples were diluted to a 10^–2^ gradient and 150 μl suspension of the dilution obtained from each rhizosphere sample was applied to Biolog EcoPlates wells. The plates were incubated at 28°C and the optical density (OD) was measured at 595 nm using a microplate absorbance spectrophotometry (Multiskan EX, Thermofisher Scientific, Waltham, MA, USA) at 24 h intervals up to 7 days incubation. Three replicates were performed for each treatment.

The microbial activity was calculated by the average well color development (AWCD) using the following equation: AWCD = Σ (C–R)/31, where C is the absorbance value of each well, and R is the absorbance value of the control (Bhattacharyya and Lee, 2016). The 31 substrates were divided into six kinds of carbon sources: phenols (pyruvic acid methyl ester, glucose phosphate, glycerol phosphate), polymers (tween 40, tween 80, cyclodextrin), carbohydrates (cellobiose, lactose, b-methyl-d-glucoside, d-xylose, erythritol, mannitol), carboxylic acids (glucosaminic acid, b-galactonic acid, galacturonic acid, hydroxybenzoic acid, 4 hydroxybenzoic acid, hydroxybutyric acid, itaconic acid, keto butyric acid, malic acid), amino acids (L-arginine, asparagine, phenylalanine, serine, threonine. glycyl glutamic acid) and amines/amides (phenylethylamine, putrescine). The AWCD for each carbon substrate group was determined using the following formula: AWCD = Σ (C–R)/N, where C is the absorbance value of each well, R is the absorbance value of the control and N is the number of substrates in the category (Bhattacharyya and Lee, 2016; Zuluaga et al., 2021). The functional diversity of microbial communities was expressed as the substrate utilization Shannon diversity index (H), substrate utilization Shannon evenness index (E) and substrate utilization Simpson diversity index (D) (Ge et al., 2018; Koner et al., 2021). The absorbance values on the 7th day of incubation were used for the calculation of metabolic functional diversity indices.

### 2.7. Microbial community structure

Richness estimators and diversity indices were calculated with mothur. LEfSe (Linear discriminant analysis effect size) pipeline, available at http://huttenhower.sph.harvard.edu/galaxy/ was used to identify taxonomic bacterial and fungal groups that were differentially abundant (*p* < 0.05) in the untreated and *Pseudomonas sivasensis* 2RO45 treated plants at each time of the inoculation (T0, T22, and T44). This analysis was performed for the 50 most abundant OTUs.

### 2.8. Data analysis and statistics

Rarefaction curves were generated using phyloseq in R v 4.0.3. Differences in microbial alpha diversity [observed OTU richness, Shannon (H’), and Simpson index (1-D)] were estimated using Past v 3.08. Two different statistical analyses in terms of microbial alpha diversity were performed: (i) *t-test* for equal means to detect differences between non-treated and *Pseudomonas sivasensis* 2RO45 treated canola plants; (ii) one-way analysis of variance (ANOVA) followed by *post-hoc* test (Tukey’s HSD test; *p*-values ≤ 0.05) to evaluate differences in the rhizosphere of *Pseudomonas sivasensis* 2RO45 treated and untreated plants according to time. Normality and homogeneity of variance assumptions were performed by the Shapiro-Wilk and Levene’s tests. Principal coordinates analysis (PCoA) and analysis of similarities (ANOSIM) were performed using R (v 4.0.3).

## 3. Result and discussion

The bioinformatic data processing resulted in 2,198,786 and 2,121,080 high quality bacterial and fungal sequence reads, respectively. Datasets covered 16,443 bacterial and 2,760 fungal OTUs. The rarefaction curves indicated a high coverage of the estimated bacterial and fungal diversity in the canola rhizosphere samples (Supplementary Figure 2).

Different alpha biodiversity indices for bacteria, e.g., OTU richness, Shannon, and Inv Simpson significantly increased over time, both in non-treated and treated with *Pseudomonas sivasensis* 2RO45 rhizosphere samples (Supplementary Table 1). Whereas, fungal alpha-diversity based on the OTU richness and Shannon diversity index were the highest in non-treated samples on 44th day (Supplementary Table 2). Rhizosphere bacterial and fungal communities were altered by various environmental factors such as climate and soil properties (Li et al., 2021), as well as host factors such as plant genotype and plant age (Fazal et al., 2021). Our results indicated that microbial richness and diversity increase with time and plant development. Previous studies also confirmed that the abundance and structure of the soil microbial community can differ depending on the plant growth stage (Liu et al., 2015; Wang et al., 2017).

Plant growth-promoting rhizobacteria inoculants can be a suitable option to developing sustainable agriculture, reducing or even eliminating the use of agrochemicals without yield loss (Kong and Liu, 2022). Nonetheless, understanding the effect of PGPR inoculation on the structure and functional dynamics in the rhizosphere microbiome is essential for better exploitation of PGPR potential in improving plant health and fitness (Zuluaga et al., 2021; Kong and Liu, 2022). Therefore, we studied biodiversity of the microbial community in the canola rhizosphere after inoculation with *Pseudomonas sivasensis* 2RO45 which features the ability to improve canola growth.

**FIGURE 1:**
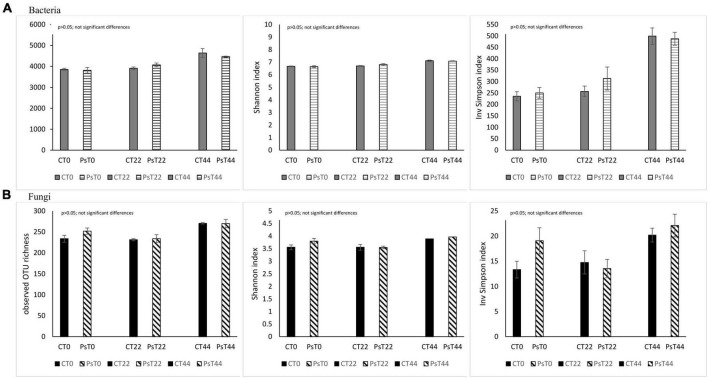
OTU richness and diversity indices (Shannon H’ and Inv Simpson) for the bacterial **(A)** and fungal **(B)** communities in *Pseudomonas sivasensis* 2RO45 treated canola rhizospheres and untreated rhizospheres samples according to treatment based on NGS sequencing; vertical bars represent standard deviation (*n* = 3).

**FIGURE 2:**
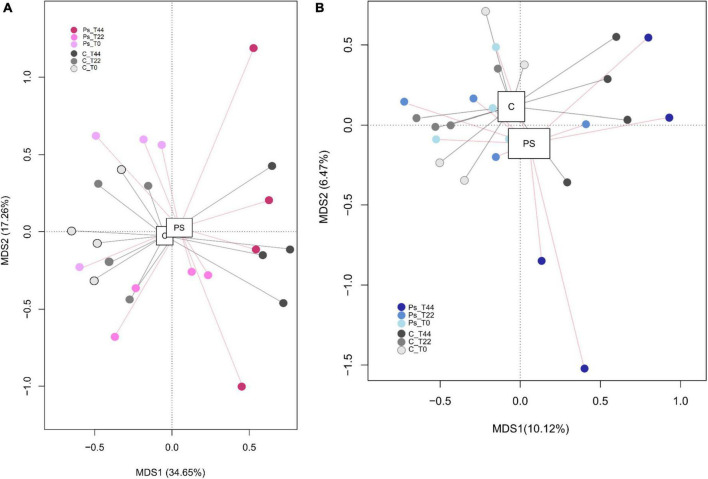
Principal coordinates analysis of bacterial **(A)** and fungal **(B)** distributions in canola plants inoculated with *Pseudomonas sivasensis* 2RO45 (PS) or uninoculated (C); T0, T22, and T44–time after inoculation in days.

There were no significant differences, given the alpha diversity metrics between non-treated and *P. sivasensis* 2RO45 treated canola plants (Figure 1), indicating that the 2RO45 strain did not significantly alter the diversity of the native soil microbiota. These results were also supported by PCoA (Figure 2) and ANOSIM (Table 1). No significant bacterial, archaeal, and diazotrophic enrichments were observed in the rhizosphere of maize (*Zea mays* L.) after *Pseudomonas stutzeri* A1501 inoculation (Ke et al., 2019). There are reports demonstrating that PGPR inoculation increases the microbial diversity in the rhizosphere, simultaneously promoting Kimchi cabbage (*Brassica rapa* L. ssp. *pekinensis*) growth (Yu and Lee, 2013; Bhattacharyya et al., 2018). However, if the PGPR inoculants are unable to survive in the competition with indigenous bacterial communities, minor or no changes would happen in terms of rhizosphere microbiome structure or diversity (Piromyou et al., 2011; Touceda-González et al., 2015). Although PGPR inoculation caused only minor changes in the rhizosphere microbial community, plant growth promotion has been observed (Piromyou et al., 2011; Touceda-González et al., 2015). Jiménez et al. (2020) showed that PGPR *Pseudomonas fluorescens* LBUM677 influences the rhizosphere microbiome of three different oilseed crops: canola (*Brassica napus* L.), soybean (*Glycine max* L.), and corn gromwell (*Buglossoides arvensis* L.). Interestingly, *P. fluorescens* LBUM677 inoculation decreased diversity of these rhizosphere microbiomes (Jiménez et al., 2020). According to Kong and Liu (2022) the effectiveness of inoculated PGPR is associated with their ability to being efficient rhizosphere colonizers. Our results showed that based on the relative abundance of the *Pseudomonas* phylotype (OTU00038) in the total bacterial community (Supplementary Figure 3) one can conclude that some *Pseudomonas* bacteria were already present in the soil before the treatment, and the inoculation of *Pseudomonas sivasensis* 2RO45 did not necessarily cause the substantial proliferation of this bacteria in the soil. Moreover, colonization of the inoculated *Pseudomonas sivasensis* 2RO45 in the rhizosphere in sterile conditions, measured as CFU was determined. The results showed that the strain was able to survive up to 44 days (Supplementary Table 3). The viability of the strain ranged from 6.6 × 10^6^ CFU/ml at T0 to 5.5 × 10^6^ CFU/ml at T44. Nevertheless, according to Chen et al. (2022), neither the colonization of roots by PGPR inoculants, nor modifications in the rhizosphere microbiome were necessary for the plant growth promotion process. The authors demonstrated that PGPR-induced DNA methylation modifications in roots mediated the long-term impact on plant growth promotion (Chen et al., 2022).

**TABLE 1:** Analysis of similarities (ANOSIM) for microbial communities in the canola rhizosphere.

Time	ANOSIM statistic R	Significance
**Bacteria**		
T0	0.219	0.100
T22	0.208	0.113
T44	0.083	0.216
**Fungi**		
T0	0.073	0.461
T22	0.042	0.443
T44	0.198	0.150

**FIGURE 3:**
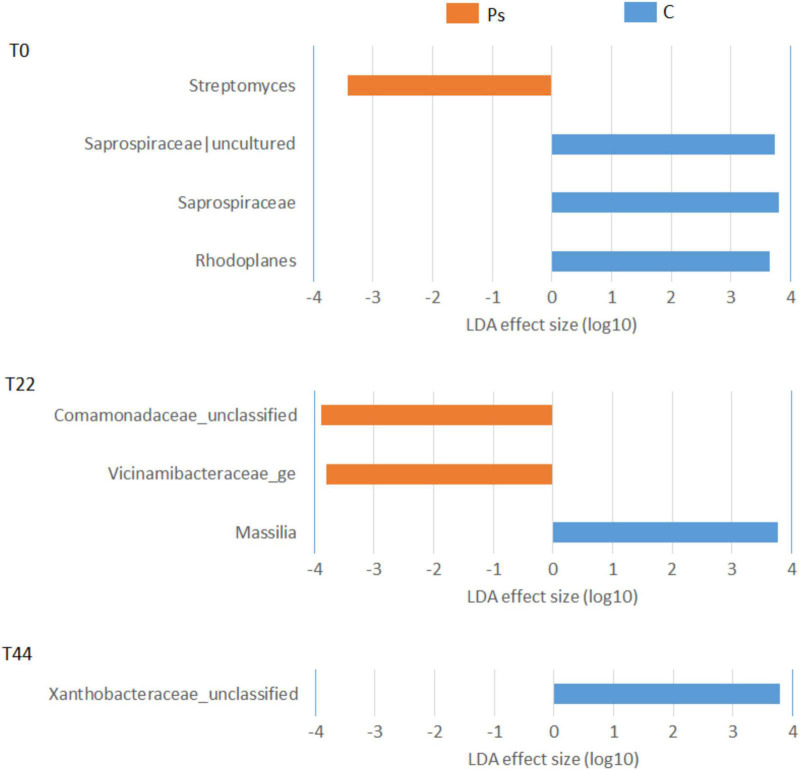
Linear discriminant analysis effect size (LEfSe) analysis showing bacterial taxonomy changes between canola plants inoculated with *Pseudomonas sivasensis* 2RO45 (PS) and uninoculated (C); T0, T22, and T44–time after inoculation in days.

**FIGURE 4:**
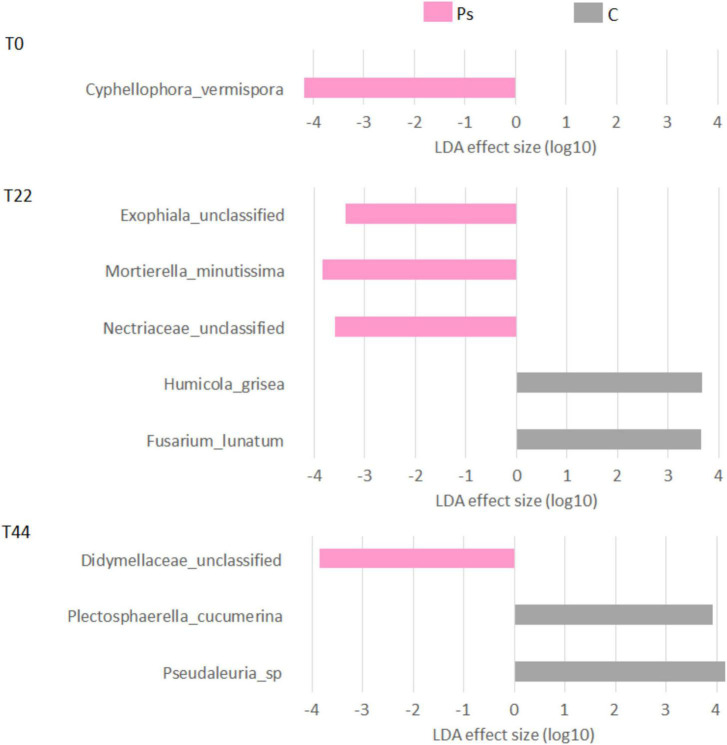
Linear discriminant analysis effect size (LEfSe) analysis showing fungal taxonomy changes between canola plants inoculated with *Pseudomonas sivasensis* 2RO45 (PS) and uninoculated (C); T0, T22, and T44–time after inoculation in days.

*Pseudomonas sivasensis* 2RO45 treatment did not alter the overall microbial diversity, but changed the proportions of some bacterial (Figure 3) and fungal (Figure 4) taxa. The introduced strain modified microbial communities, particularly increasing the abundance of microorganisms beneficial for a plant growth. Initially at T0, *P. sivasensis*, 2RO45 increased the abundance of genus *Streptomyces*, then at T22 bacterial families *Comamonadaceae* and *Vicinamibacteraceae* were more abundant in the *P. sivasensis* 2RO45 treated soil. Whereas, at T44 *P. sivasensis* 2RO45 strain did not cause any changes in the bacterial community structure. Numerous studies reported that *Streptomyces* has beneficial associations with plants, improving their growth and protecting them against bacterial and fungal diseases through the production of antibiotics and bioactive compounds (Amaresan et al., 2018; Suárez-Moreno et al., 2019; Vergnes et al., 2020; Le et al., 2021). Bacteria belonging to the *Vicinamibacteraceae* family are degraders of organic matter and chitin (Whitton et al., 2022), while members of the family *Comamonadaceae* are able to control *Fusarium* wilt disease by secreting more organic acids (Wen et al., 2020). The changes in taxonomic structure until 22 days after *P. sivasensis* 2RO45 bacterization indicated a transient and short-term perturbation in the taxonomic structure of bacterial communities. The results also indicate that the taxonomic structure of bacterial communities in young canola plants was more dynamically influenced by *P. sivasensis* 2RO45 bacterization compared to that in older plants. Similar results were obtained by Bhattacharyya and Lee (2016).

After *P. sivasensis* 2RO45 inoculation into the soil, the highest changes in fungal communities in comparison to the control were observed at T22. Initially at T0, *Cyphellophora vermispora* was more abundant in *P. sivasensis* 2RO45 treated soil, then at T22 *P. sivasensis* 2RO45 increased the abundance of the fungal family of *Nectriaceae*, genus *Exophiala* and species *Mortierella minutissima*. Whereas, at T44 *P. sivasensis* 2RO45 increased the abundance of the *Didymellaceae* family. Species of *Didymellaceae* were found as plant fungal pathogens causing fruit, leaf, stem, and root lesions on a wide variety of crops (Hou et al., 2020). However, *Didymellaceae* is the largest family within the order *Pleosporales* with more than 5 400 taxon names, including also saprobic, endophytic, and clinically relevant species (Hou et al., 2020; Yuan et al., 2021). *Exophiala* has been effectively used in a agricultural biotechnology. For instance, *Exophiala pisciphila* stimulated maize growth (Xu et al., 2020) and suppressed Fusarium-wilt disease in strawberries (Harsonowati et al., 2020), while *Exophiala* sp. promoted cucumber growth under abiotic stresses (Khan et al., 2011). *Cyphellophora vermispora* was isolated from a natural environment such as plant stems, roots, and leaves (Gao et al., 2015). Members of the *Nectriaceae* family have been reported as plant opportunistic pathogens; however, several species belonging to the *Nectriaceae* have been also used as biocontrol agents and biodegraders for developing sustainable agriculture (Lombard et al., 2015). The genus *Mortierella* includes numerous PGP fungi degrading biopolymers, e.g., *M. minutissima* isolated from the root surface had a strong chitinolytic activity (Ozimek and Hanaka, 2020).

The community-level physiological profiling (CLPP) using Biolog EcoPlates was estimated to analyze the changes in the canola rhizosphere metabolic profile and functional diversity in response to *P. sivasensis* 2RO45 introduction. The alterations in the metabolic profile of microbial communities from non-treated and *P. sivasensis* 2RO45 treated rhizospheres were analyzed on the 44th day. This time point was chosen to reveal if *P. sivasensis* 2RO45 inoculation has a long-term impact on the functional diversity of the canola rhizosphere microbiome.

The metabolic functional diversity indices, except substrate utilization Simpson index (D), had significant differences (*p* < 0.05) between non-treated and *P. sivasensis* 2RO45 treated canola rhizospheres (Table 2). Substrate utilization Shannon diversity (H) index and evenness (E) were the highest in canola plants inoculated with *P. sivasensis* 2RO45, indicating that the functional diversity of the canola rhizosphere microbial community was altered by bacterization. The changes in the metabolic profiles of soil microbial communities after PGPR introduction is in line with previous studies on plants, such as wheat (Di Salvo et al., 2018b), maize (Di Salvo et al., 2018a), tomato (Zuluaga et al., 2021), and rice (de Salamone et al., 2010).

**TABLE 2:** Functional diversity indices based on Biolog EcoPlates results on the 7th day of incubation for the canola rhizosphere microbial communities.

Sample	Shannon diversity index (H)	Shannon evenness index (E)	Simpson diversity index (D)
C	3.01 ± 0.05*	0.89 ± 0.02*	0.92 ± 0.01
Ps	3.12 ± 0.03*	0.91 ± 0.01*	0.92 ± 0.01

Ps–canola plants inoculated with *Pseudomonas sivasensis* 2RO45, C–control, uninoculated canola plants; significant differences (*p < 0.05) according to *t*-test.

**FIGURE 5:**
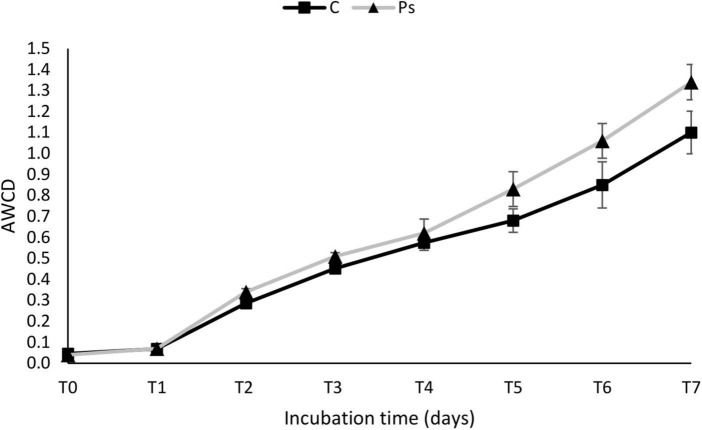
The average well color development (AWCD) of all carbon sources in *Pseudomonas sivasensis* 2RO45 treated and non-treated rhizosphere samples according to incubation time; vertical bars represent standard deviation (*n* = 3).

**FIGURE 6:**
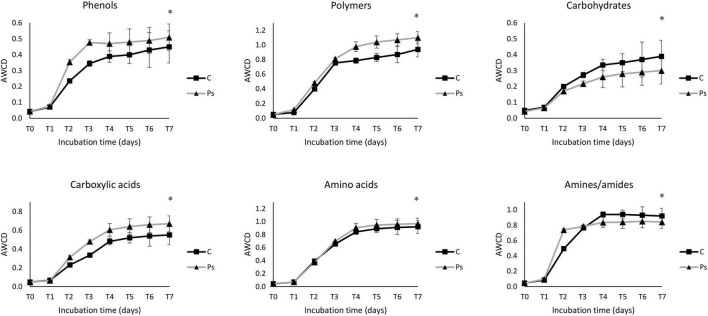
The average well color development (AWCD) of six carbon source groups, including phenols, polymers, carbohydrates, carboxylic acids, amino acids, and amines/amides in *Pseudomonas sivasensis* 2RO45 treated and non-treated rhizosphere samples; vertical bars represent standard deviation (*n* = 3); significant differences (*p < 0.05).

The average well color development (AWCD) curve of all carbon sources was plotted to check the metabolic activity of microbial communities (Figure 5). The microbial communities of the *P. sivasensis* 2RO45 treated canola rhizospheres samples exhibited higher AWCD values than the control samples, suggesting that microbiota associated with *P. sivasensis* 2RO45 were more active in their use of different types of carbon substrates during cell growth than the indigenous microbiota and the overall metabolic activity was increased by bacterization. Furthermore, the microbial activity in the canola rhizospheres was calculated by AWCD for six categories of carbon sources: phenols, carbohydrates, amino acids, carboxylic acids, polymers, and amines (Figure 6). The microbial communities in the *P. sivasensis* 2RO45 treated canola rhizospheres samples were found to utilize phenols, polymers, carboxylic acids, and amino acids more intensively than those in the non-treated samples. Whereas, carbohydrates and amines were better metabolized by the rhizosphere community from non-treated canola plants. The results are similar to the findings reported by previous research, where the metabolic profile of microbial communities was modified due to PGPR bacterization (Bhattacharyya and Lee, 2016; Zuluaga et al., 2021). According to Bhattacharyya and Lee (2016), an increase in the utilization of amino acids and a reduction in the utilization of carbohydrates in the bacterized rhizosphere could have a beneficial effect on Kimchi cabbage growth. Whereas, Zuluaga et al. (2021) found that carbohydrates, amines, polymers, and phenolic compounds were the main carbon substrate groups that contribute to the rhizosphere microbiome function between rhizosphere plants inoculated with PGPR *Pseudomonas* sp. and non-inoculated plants. The authors suggested that rhizobacterial inoculants can modulate the rhizosphere microbiome function by affecting the root exudation profile, consequently interfering in the plant-soil feedback and the shaping of the plant-associated microbial communities (Zuluaga et al., 2021).

## 4. Conclusion

Plant growth-promoting rhizobacteria *Pseudomonas sivasensis* 2RO45 inoculation altered the taxonomic structure of canola rhizosphere microbial communities by increasing the abundance of plant beneficial microorganisms: bacteria affiliated with families *Comamonadaceae*, *Vicinamibacteraceae*, genus *Streptomyces*, and fungi assigned to the *Nectriaceae, Didymellaceae, Exophiala, Cyphellophora vermispora*, and *Mortierella minutissima*. Moreover, *P. sivasensis* 2RO45 induced perturbations in the rhizosphere microbiome by increasing metabolic activity and functional diversity of microbial communities. Phenols, polymers, carboxylic acids, and amino acids were the major classes of carbon substrates that contributed to the function of the rhizosphere microbiome after inoculation with *P. sivasensis* 2RO45. The results provide new insight and future perspectives into PGPR-canola interactions for sustainable agriculture development. The introduction of *P. sivasensis* 2RO45 is beneficial to development of a sustainable agriculture because it changes the native microbiota crucial for the proper functioning of the soil. The minor changes that are observed are positive, especially the increase of metabolic activity which may increase the possibility of detoxification of the environment (decomposition of phenols and polymers) or the elements circulation (e.g., carbon and nitrogen).

## Data availability statement

The datasets presented in this study can be found in online repositories. The names of the repository/repositories and accession number(s) can be found in the article/Supplementary material.

## Author contributions

JŚ wrote the original draft, made greenhouse experiment and sampling, made isolation of DNA, made community-level physiological profiles (CLPP) analysis using Biolog EcoPlates, conducted statistical analyses, and prepared the manuscript editorially. AK made rarefaction curves, LEfSe PCoA, and ANOSIM analysis, coordinated the study, and checked the validity of the original draft. AS carried out the sequence analysis. MS designed and coordinated the study and checked the validity of the original draft. All authors contributed to the article and approved the submitted version.
