# Supplementary material for: Pseudomonas sivasensis 2RO45 inoculation alters the taxonomic structure and functioning of the canola rhizosphere microbial community
Source: Front Microbiol. 2023 May 5;14:1168907. doi: 10.3389/fmicb.2023.1168907 (PMC10196004; doi:10.3389/fmicb.2023.1168907)
Supplement: Supplementary file 1 [file Data_Sheet_1.docx]

**Table S1** OTU richness and diversity indices (Shannon H’ and Inv Simpson) for the bacterial community in *Pseudomonas* *sivasensis* 2RO45 treated canola rhizospheres and untreated rhizospheres samples according to time based on NGS sequencing.

| **Sample** | **Observed OTU richness** | **Mean/**  **Median** | ***P*-value** | **Shannon**  **H’** | **Mean** | ***P*-value** | **Inv**  **Simpson** | **Mean/**  **Median** | ***P*-value** |
| --- | --- | --- | --- | --- | --- | --- | --- | --- | --- |
| CT0_1 | 3807.99 | 3834.69^nnd^ | 0.02 | 6.62 | 6.66 | 0.00 | 202.69 | 235.76 | 0.00 |
| CT0_2 | 3861.39 |  |  | 6.66 |  |  | 233.10 |  |  |
| CT0_3 | 3745.34 |  |  | 6.63 |  |  | 214.20 |  |  |
| CT0_4 | 4009.30 |  |  | 6.75 |  |  | 293.04 |  |  |
| CT22_1 | 3884.38 | 3907.03^nnd^ |  | 6.63 | 6.70 |  | 217.32 | 257.16 |  |
| CT22_2 | 4075.07 |  |  | 6.79 |  |  | 316.38 |  |  |
| CT22_3 | 3929.69 |  |  | 6.72 |  |  | 272.67 |  |  |
| CT22_4 | 3743.00 |  |  | 6.64 |  |  | 222.25 |  |  |
| CT44_1 | 5082.35 | 4682.86^nnd^ |  | 7.22 | 7.11 |  | 556.83 | 499.29 |  |
| CT44_2 | 4866.79 |  |  | 7.18 |  |  | 555.09 |  |  |
| CT44_3 | 4498.94 |  |  | 7.09 |  |  | 480.03 |  |  |
| CT44_4 | 4127.62 |  |  | 6.95 |  |  | 405.20 |  |  |
| PsT0_1 | 3750.54 | 3813.61 | 0.00 | 6.60 | 6.65 | 0.00 | 241.66 | 257.21^nnd^ | 0.02 |
| PsT0_2 | 4033.27 |  |  | 6.78 |  |  | 299.99 |  |  |
| PsT0_3 | 4012.88 |  |  | 6.75 |  |  | 272.77 |  |  |
| PsT0_4 | 3457.76 |  |  | 6.47 |  |  | 188.80 |  |  |
| PsT22_1 | 4261.21 | 4067.82 |  | 6.94 | 6.81 |  | 406.15 | 321.70^nnd^ |  |
| PsT22_2 | 4057.99 |  |  | 6.75 |  |  | 250.91 |  |  |
| PsT22_3 | 3836.12 |  |  | 6.64 |  |  | 204.59 |  |  |
| PsT22_4 | 4115.96 |  |  | 6.91 |  |  | 392.48 |  |  |
| PsT44_1 | 4490.12 | 4468.30 |  | 7.13 | 7.09 |  | 530.12 | 485.90^nnd^ |  |
| PsT44_2 | 4479.74 |  |  | 7.14 |  |  | 541.79 |  |  |
| PsT44_3 | 4399.89 |  |  | 7.05 |  |  | 441.68 |  |  |
| PsT44_4 | 4503.44 |  |  | 7.03 |  |  | 436.80 |  |  |

nnd – not normal distribution (test for equal medians: Kruskal-Wallis); ns – non significant; variants marked with different letters indicate groups signiﬁcantly different from each other, p<0.05 (Tukey's test as post hoc); C – control, untreated plants; Ps – plants treated with *Pseudomonas sivasensis* 2RO45; T0, T22 and T44 - time after bacterization in days.

**Table S2** OTU richness and diversity indices (Shannon H’ and Inv Simpson) for the fungal community in *Pseudomonas* *sivasensis* 2RO45 treated canola rhizospheres and untreated rhizospheres samples according to time based on NGS sequencing.

| **Sample** | **Observed OTU richness** | **Mean/**  **Median** | ***P*-value** | **Shannon**  **H’** | **Mean** | ***P*-value** | **Inv**  **Simpson** | **Mean/**  **Median** | ***P*-value** |
| --- | --- | --- | --- | --- | --- | --- | --- | --- | --- |
| CT0_1 | 219.58 | 231.89^nnd^ | 0.02 | 3.48 | 3.55 | 0.02 | 12.30 | 13.34 | ns |
| CT0_2 | 251.54 |  |  | 3.72 |  |  | 15.76 |  |  |
| CT0_3 | 243.26 |  |  | 3.70 |  |  | 16.20 |  |  |
| CT0_4 | 220.52 |  |  | 3.31 |  |  | 9.12 |  |  |
| CT22_1 | 233.55 | 230.59^nnd^ |  | 3.57 | 3.55 |  | 14.89 | 14.76 |  |
| CT22_2 | 227.63 |  |  | 3.78 |  |  | 21.24 |  |  |
| CT22_3 | 227.61 |  |  | 3.38 |  |  | 11.49 |  |  |
| CT22_4 | 237.23 |  |  | 3.48 |  |  | 11.43 |  |  |
| CT44_1 | 268.94 | 269.29^nnd^ |  | 3.89 | 3.89 |  | 20.81 | 20.22 |  |
| CT44_2 | 276.08 |  |  | 4.02 |  |  | 23.66 |  |  |
| CT44_3 | 267.78 |  |  | 3.77 |  |  | 17.09 |  |  |
| CT44_4 | 269.65 |  |  | 3.87 |  |  | 19.30 |  |  |
| PsT0_1 | 254.01 | 251.90 | ns | 3.80 | 3.80 | ns | 17.64 | 19.06 | ns |
| PsT0_2 | 271.84 |  |  | 4.11 |  |  | 26.80 |  |  |
| PsT0_3 | 243.99 |  |  | 3.64 |  |  | 15.94 |  |  |
| PsT0_4 | 237.78 |  |  | 3.66 |  |  | 15.88 |  |  |
| PsT22_1 | 260.56 | 234.17 |  | 3.81 | 3.55 |  | 17.66 | 13.54 |  |
| PsT22_2 | 230.43 |  |  | 3.66 |  |  | 15.49 |  |  |
| PsT22_3 | 226.00 |  |  | 3.40 |  |  | 11.29 |  |  |
| PsT22_4 | 219.70 |  |  | 3.34 |  |  | 9.74 |  |  |
| PsT44_1 | 286.28 | 270.00 |  | 4.07 | 3.96 |  | 24.92 | 22.10 |  |
| PsT44_2 | 287.76 |  |  | 4.14 |  |  | 26.73 |  |  |
| PsT44_3 | 255.85 |  |  | 3.87 |  |  | 19.98 |  |  |
| PsT44_4 | 250.10 |  |  | 3.77 |  |  | 16.78 |  |  |

nnd – not normal distribution (test for equal medians: Kruskal-Wallis); ns – non significant; variants marked with different letters indicate groups signiﬁcantly different from each other, p<0.05 (Tukey's test as post hoc); C – control, untreated plants; Ps – plants treated with *Pseudomonas sivasensis* 2RO45; T0, T22 and T44 - time after bacterization in days.

**Table S3** *Pseudomonas sivasensis* 2RO45 load at different time.

| **Time** | ***Pseudomonas sivasensis* 2RO45 load (x 10^6^ CFU/ml)** |
| --- | --- |
| T0 | 6.6 |
| T22 | 5.7 |
| T44 | 5.5 |


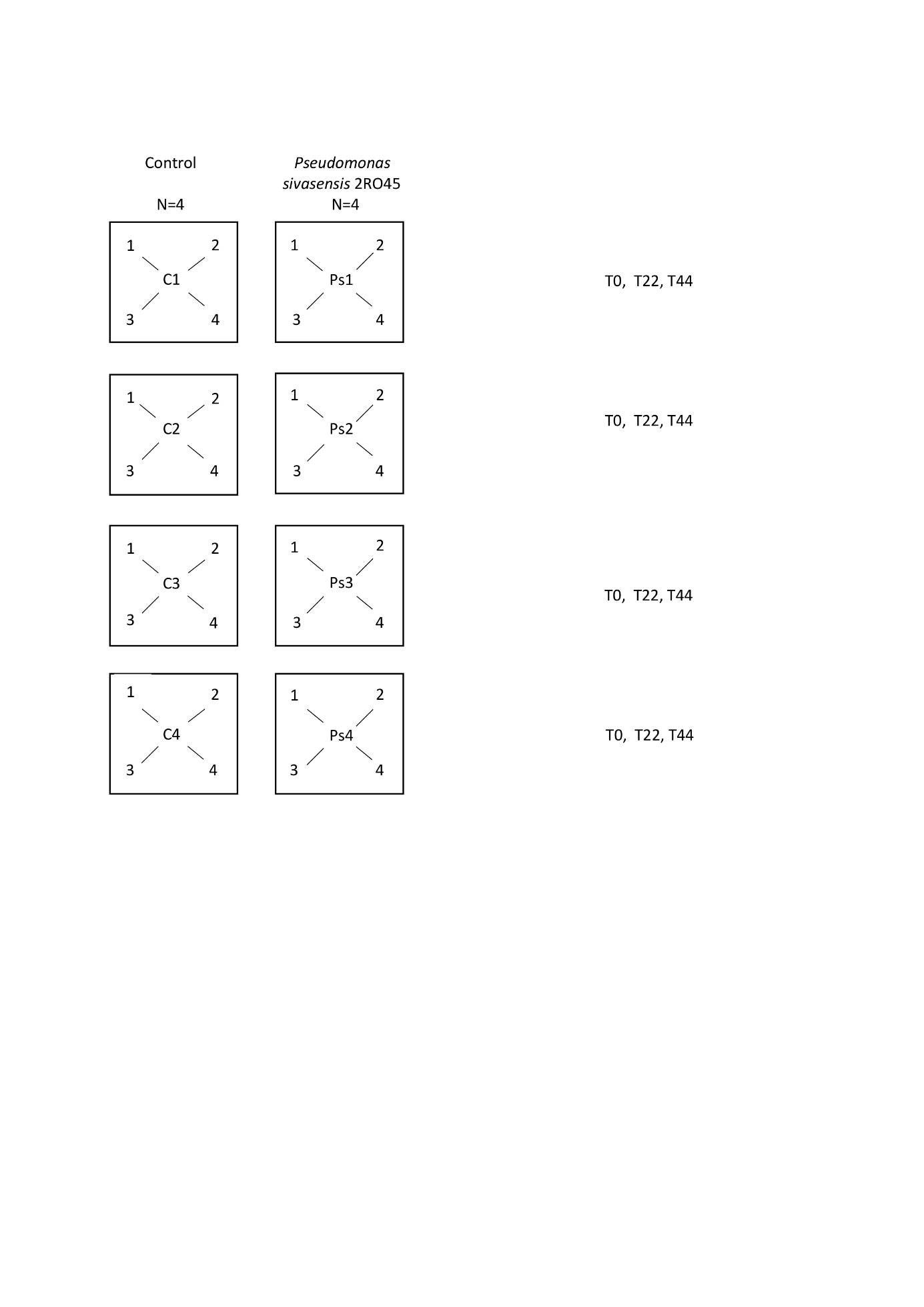


**Figure S1** Canola rhizosphere sampling procedure. Pooled samples (N=4) were taken from the canola rhizosphere at the time of introducing the *Pseudomonas sivasensis* 2RO45 (T0), after 22 days (T22), and after 44 days (T44).

**
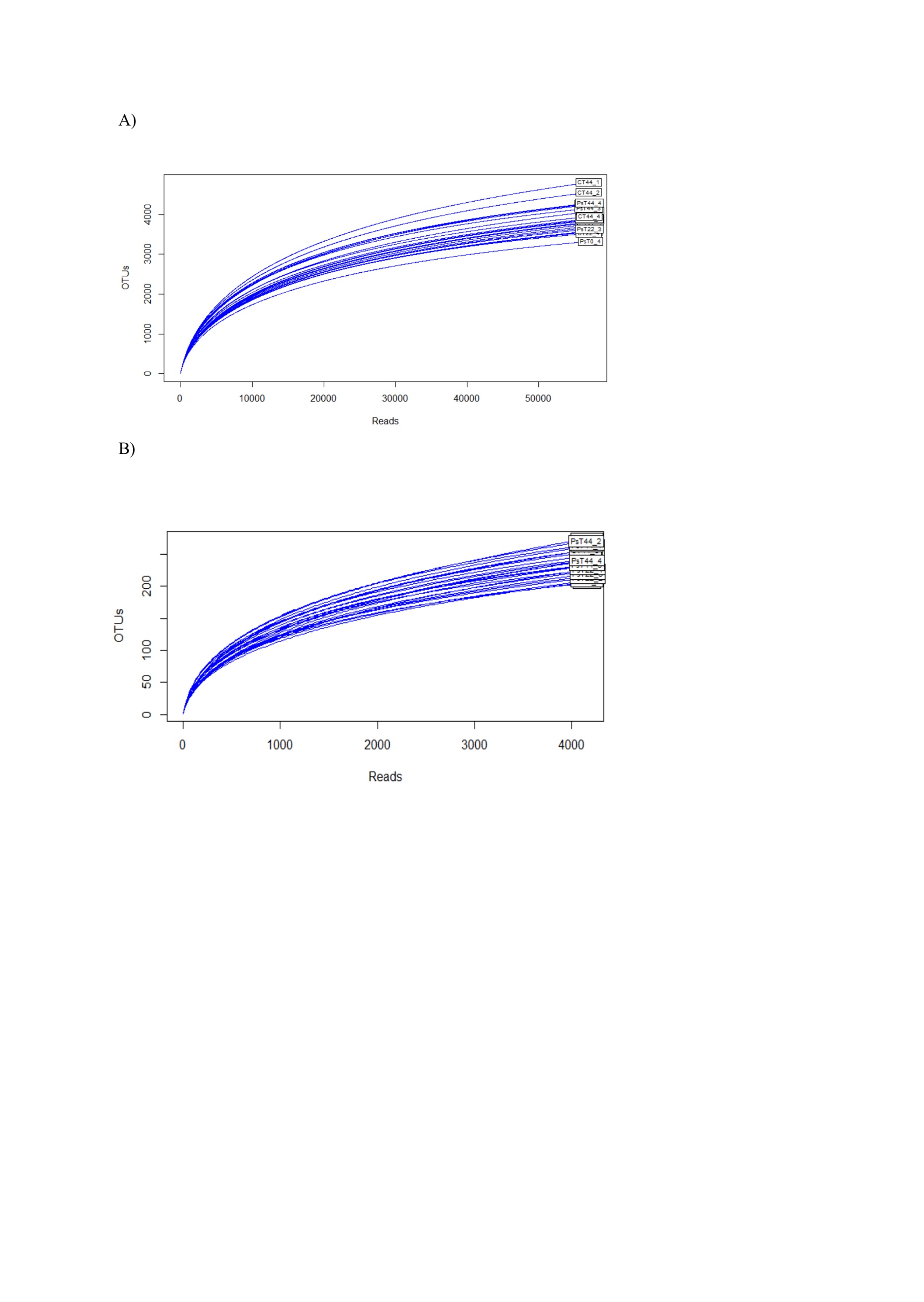
**

**Figure S2** Rarefaction curves for the (A) bacterial (B) fungal soil 16S rRNA gene sequences.


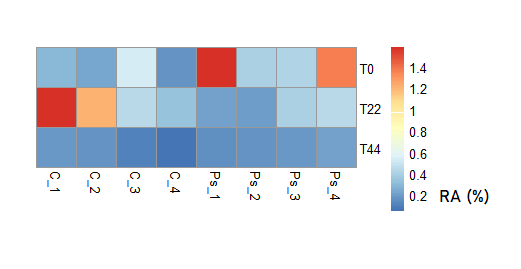


**Figure S3** Relative abundance (%) of *Pseudomonas* phylotype (OTU00038) in total bacterial community.
